# The X Ray

**Published:** 1896-05

**Authors:** 


### THE X-RAY.

The X-ray is a peculiar emanation from strong electrical discharges through glass tubes exhausted of air up to 1-200,000, to 1–400,000 of its ordinary density. These tubes are known as Crooke's or Geissler's tubes. Into opposite ends of the tube metallic pieces are hermetically fastened and to these are attached the ends of the wire of a strong induction coil. When a current is passed through the coil, discharges pass from one piece of metal to the other, lighting up the tube and developing in its glass walls a bright fluorescence. It is from the fluorescent walls the peculiar emanation or X-ray radiates. Whilst invisible to the eye, they are called rays, because like light, they produce shadows on photographic or other sensitive plates; develop chemical changes and decrease in intensity inversely as the squares of the distances. Whilst thus having some of the properties of light, these have not as yet been identified with any of the known forms of light—neither its visible or invisible rays. They differ from the light of the electric discharges (cathode rays) in not being deflected by a magnet (which the cathode rays are), and from sunlight in the absence of illuminating power, in passing ough many substances entirely opaque to sunlight, and in suffering no refraction in passing from one medium into another. From a medical standpoint, interest in the X-ray arises from three things. First, its ability to penetrate a great many substances opaque to ordinary light, and its inability to penetrate others. Second, its power to produce changes in sensitive photographic plates, hence power to produce a certain kind of photograph. Third, its power to produce fluorescence in certain substancs, as the double cyanide of platinum and barium, sulphide of calcium, etc.

First, as to penetrating power. It will affect a photographic plate after passing rough black pasteboard, a thick book, a thick plank of wood, through flesh the thickness of that in the hand or arm and through cartilage. It will not pass through most metals, unless in very thin plates, with the exception of aluminum, through which it passes quite readily. Lead is especially opaque to it, so is bone. It will be seen, therefore, that if a piece of metal or bone be placed between a tube from which the rays emanate and a photographic plate, the ray not passing through metal or bone, a shadow of these will be made on the plate, and then the latter is developed chemically, the shadow will be shown, giving the outlines of the metal or bone. Such pictures are called shadowgraph, skotograph, skiagraph, etc. The sensitive plate is placed in the usual box and the object to be photographed placed upon, or between it and the Crooke's tube; the sides of the box being readily penetrated by the X-ray. The photographing may be dispensed with however, and the shadow of an object seen directly with the eye by means of an instrument called a cryptoscope or skiascope. This consists of a tube three or four inches long and of diameter to suit the size of the object to be examined. It is blackened on the inside; at one end is fitted a piece of sensitive paper (paper on which a paste of double cyanide of platinum and barium has been spread) with the paste on the inside. The other end is fitted to the eye; a lens could be fitted to the eye end if desired. Now the object to be examined being placed between the Crooke's excited tube and skiascope, if any substance opaque to the X-ray be in it, a shadow of it will fall on the sensitive paper; all around the shadow the sensitive paper will be made bright and fluorescent by the X-rays, whilst the shadow will remain dark and therefore the outline of the opaque object be distinctly visible to the eye. To just what uses the X-ray will be of service in medicine and surgery, remains to be seen; of course, the various methods of applying it will be gradually perfected and much that is now obscure will be made plain. The detection of foreign metallic bodies in the body, of exostoses, fractured bones, etc., are among its probable early application.

---

### SOUTHERN RAILWAY COMPANY. ALABAMA GREAT SOUTHERN RAILROAD COMPANY.

OFFICE OF CHIEF SURGEON, ATLANTA, GA., April 4th, 1896.

*To All Company Surgeons:*

The American Medical Association will meet in Atlanta, Tuesday, May 5th, next, and the American Academy of Medicine will convene in the same city, Saturday, May 2nd. As many of the Surgeons of the Southern Railway and the Alabama Great Southern Railroad will be in attendance upon these interesting meetings, it is deemed advisable to invite all Surgeons of these Companies to meet in Atlanta on Monday morning, May 4th, 1896, for the purpose of adopting permanent organization of a Railway Association composed of Surgeons of the two Systems. The object of such an organization will be to promote acquaintance and friendly relations among the Corps; to bring about a free interchange of ideas and discussions of railway surgery and its further development.

Believing that such a meeting will be productive of much good in the advancement of railway surgery, and impressed with a full sense of the mutual benefits to be derived by Surgeons of the Company through such an intercourse, I hereby invite all Surgeons of the Southern Railway Company and the Alabama Great Southern Railway Company, who can be present, to meet in the ballroom of the Kimball House, Atlanta, Ga., Monday, May 4th, 1896, at 9.00 a. m.

Important papers on Railway Surgery will be presented for discussion. All surgeons of the Companies are earnestly invited to be present and to read papers bearing on Railway Surgery. Kindly send titles of papers to the Chief Surgeon on or before April 25th, so that a full programme can be printed and distributed. Several Surgeons of prominence have already signified their willingness to present papers at this meeting. Important matters pertaining to the Surgical Department of the two Systems of Railroads will be presented at the meeting by the Chief Surgeon. Printed programmes will be sent to each Surgeon about May 1st.

Arrangements for reduced rates at the Kimball House and Aragon hotel have been effected.

Applications for transportation of Company Surgeons and members of their families should be made to Chief Surgeon.

All communications addressed to this office will receive prompt attention and response.

Respectfully,

C. M. DRAKE, Chief Surgeon.

---

The following announcement has been sent out by Dr. Drake, Chief Surgeon of the Southern Railway.

SOUTHERN RAILWAY COMPANY,
OFFICE OF CHIEF SURGEON.
C. M. DRAKE, CHIEF SURGEON.

ATLANTA, GA., April 23, 1896.

*To Members of the American Medical Association:*

The Chief Surgeon of the Southern Railway Company begs to announce to members of the American Medical Association that the Southern Railway Company has the pleasure of extending to the members and their families, a complimentary excursion, on Wednesday, May 6, 1896, from Atlanta to Lithia Springs, Georgia, and return, on the occasion of an old-fashioned "Georgia Barbecue" to be given at the Springs on that date by Mr. Marsh, under the supervision of the Committee of Arrangements of the American Medical Association.

The Chief Surgeon of the Southern Railway Company also has the pleasure of announcing a complimentary journey to be extended to a limited number of distinguished members of the American Medical Association, to leave Atlanta on the evening of Friday, May 8, to visit Lookout Mountain and Tate Epsom Springs, Tennessee, Hot Springs, Asheville, and the "Land of the Sky," Western North Carolina, returning to Atlanta.

Invitations to this excursion will be issued to members of this Association after registration with the Permanent Secretary.

Guests are advised that the itinerary of this journey contemplates a connection at Salisbury, North Carolina, with the east-bound vestibule train for Washington, New York and points East.

For this excursion Pullman Sleeping cars and other accommodations will be supplied for the invited guests, and illustrated souvenir pamphlets will be furnished, giving full description of the points to be visited.

Yours truly, C. M. DRAKE, Chief Surgeon.

---

THE Seventeenth Annual report of the Board of Health of the City of Atlanta, for the year 1895, is just out. The report contains much of interest to the physician, and gives valuable statistics. Accompanying the report is a map of the city of Atlanta, showing by different colored markings, the localities where diphtheria, scarlet fever and *fatal* cases of typhoid fever occurred during the year 1895. The map also shows where the drinking supply of water was from wells. Such work is in the right direction, and if persevered in by the Board, will in time, accomplish much good.

We further note that the city ordinance requiring physicians to report contagious diseases, has been so amended as to include in the list typhoid fever and measles, and now reads:

"It shall likewise be the duty of the attending physician to give immediate notice to the Board of Health Office of any contagious or infectious disease of any character, such as Small-Pox, Cholera, Diphtheria, Membranous Croup, Measles, Typhoid Fever, Typhus Fever, Scarlet Fever, Yellow Fever, and such other diseases as may be declared, by said Board of Health, to be contagious or infectious, which may come under the professional care of such physician; and it shall also be the duty of attending physician to report to the Board of Health Office when such patients are free from contagion and house ready for disinfecting and fumigating; and any physician who shall fail or neglect to report, as aforesaid, any such cases of diseases that come under his professional care, as aforesaid, shall be punished, on conviction thereof in Recorder's Court, by fine not to exceed $100 and cost, or imprisonment not to exceed thirty days, either or both in the discretion of the Court."

